# TiO_2_/SnO_2_ Bilayer Electron Transport Layer for High Efficiency Perovskite Solar Cells

**DOI:** 10.3390/nano13020249

**Published:** 2023-01-06

**Authors:** Xiaolin Sun, Lu Li, Shanshan Shen, Fang Wang

**Affiliations:** 1 School of Aeronautical Engineering, Nanjing Vocational University of Industry Technology, Nanjing 210046, China; 2 School of Electrical Engineering, Nanjing Vocational University of Industry Technology, Nanjing 210046, China; 3 College of Electronic Engineering, Nanjing XiaoZhuang University, Nanjing 211100, China

**Keywords:** perovskite solar cells, electron transport layer, low hysteresis, SnO_2_, TiO_2_

## Abstract

The electron transport layer (ETL) has been extensively investigated as one of the important components to construct high-performance perovskite solar cells (PSCs). Among them, inorganic semiconducting metal oxides such as titanium dioxide (TiO_2_), and tin oxide (SnO_2_) present great advantages in both fabrication and efficiency. However, the surface defects and uniformity are still concerns for high performance devices. Here, we demonstrated a bilayer ETL architecture PSC in which the ETL is composed of a chemical-bath-deposition-based TiO_2_ thin layer and a spin-coating-based SnO_2_ thin layer. Such a bilayer-structure ETL can not only produce a larger grain size of PSCs, but also provide a higher current density and a reduced hysteresis. Compared to the mono-ETL PCSs with a low efficiency of 16.16%, the bilayer ETL device features a higher efficiency of 17.64%, accomplished with an open-circuit voltage of 1.041 V, short-circuit current density of 22.58 mA/cm^2^, and a filling factor of 75.0%, respectively. These results highlight the unique potential of TiO_2_/SnO_2_ combined bilayer ETL architecture, paving a new way to fabricate high-performance and low-hysteresis PSCs.

## 1. Introduction

The high-efficiency, low-cost and facile fabrication process of halide perovskite solar cells (PSCs) have attracted tremendous attention in the field of photovoltaics in the past decade [1,2,3,4,5] and been regarded as the most promising substitute for traditional silicon (Si) and copper indium gallium selenide (CIGS) solar cells [6,7,8]. The sandwich structure of hybrid organic-inorganic based PSCs includes the electron transport layer (ETL), perovskite absorber layer, hole transport layer (HTL) and electrodes. Among them, ETL and HTL are used for the electron and hole extraction, respectively. However, the Spiro-OMeTAD are widely used as HTL in PSCs because of the simple synthesis, high carrier mobility and suitable valance band. The HTL are always fabricated by spin-coating on the top of a perovskite absorber layer with a dense and uniform film. In contrast, the ETL in PSCs is usually fabricated in a planar and/or mesoporous structure under the perovskite absorber layer [9,10,11]. The surface quality of ETL can substantially influence the deposition of perovskite film. Therefore, the electron transport layer and the corresponding interface of ETL/perovskite are significantly important parts to fabricate high-quality PSCs. Titanium dioxide (TiO_2_) and/or tin oxide (SnO_2_) thin films have been extensively investigated as an effective ETL in the PSCs, which can be fabricated by several different methods such as spin-coating, sputtering and chemical bath deposition (CBD) [12,13,14,15,16], to pursue a higher performance device.

Due to the facile planar configuration of PSCs, fabricating uniform, and compact ETL thin layer, it is imperative to pursue high performance. The conventional spin-coating method shows a facile and efficient way to fabricate the TiO_2_-ETL. However, the uneven distribution of TiO_2_ nanoparticles result in the carrier accumulation between perovskite (PVSK) and the ETL interface and an insufficient carrier extraction, leading to a low efficiency of resultant device [17,18]. Moreover, the large hysteresis of TiO_2_-ETL also impedes the further application of TiO_2_ in the PSCs [19]. Alternatively, SnO_2_ presents a reduced hysteresis, high carrier mobility and good energy level towards perovskite, which can greatly improve the performance of PSCs [20,21,22]. For example, You et al. proposed SnO_2_ as a planar ETL in the PSCs, which not only reduces the energy barrier between ETL/PVSK, but also reduces the hysteresis of devices, resulting in a high performance PSC with a champion PCE of 20.5% [21]. However, uniformity of SnO_2_ nanoparticles is still a concern for the device fabrication because of its uneven distribution by spin-coating technique. Therefore, high-quality ETL plays a crucial role in the fabrication of devices, which paves a promising way for high-efficiency PSCs. To address this issue, Xu et al. introduced a bilayer ETL of TiO_2_/ZnO thin layers into PSCs, which produces a compact interfacial layer to avoid direct contact between the FTO substrate and PVSK, leading to a reduced carrier accumulation at ETL/PVSK interface [23].

In this work, we propose a bilayer of ETLs that is composed of a CBD TiO_2_ layer and a spin-coated SnO_2_ layer. The presence of the SnO_2_ thin layer on the top surface of CBD TiO_2_ film can provide a higher current density and reduce the hysteresis of PSCs simultaneously. In addition, the diffusion of the K ion from SnO_2_ can significantly improve the crystallinity of grains in the perovskite films. On the basis of this bilayer strategy, a higher power conversion efficiency (PCE) of 17.64% was achieved in comparison with the mono-TiO_2_ ETL based PSCs with a PCE of 16.16%.

## 2. Materials and Methods

Materials: All reagents were used as received without further purification. Methylammonium iodide (MAI), methylammonium bromide (MABr), methylammonium chloride (MACl), formamidinium iodide (FAI), lead(II) iodide (PbI_2_) and 2,2′,7,7′-tetrakis(N,N-di-p-methoxyphenylamine)9,9′-spirobifluorene (Spiro-OMeTAD) (99.5%) were purchased from Xi’an Polymer Light Technology (Xi’an, China). Dimethylformamide (DMF), dimethyl sulfoxide (DMSO), isopropanol (IPA), chlorobenzene (CB), and titanium tetrachloride (TiCl_4_) were purchased from Sigma-Aldrich (Milwaukee, Germany).

**Figure 1:**
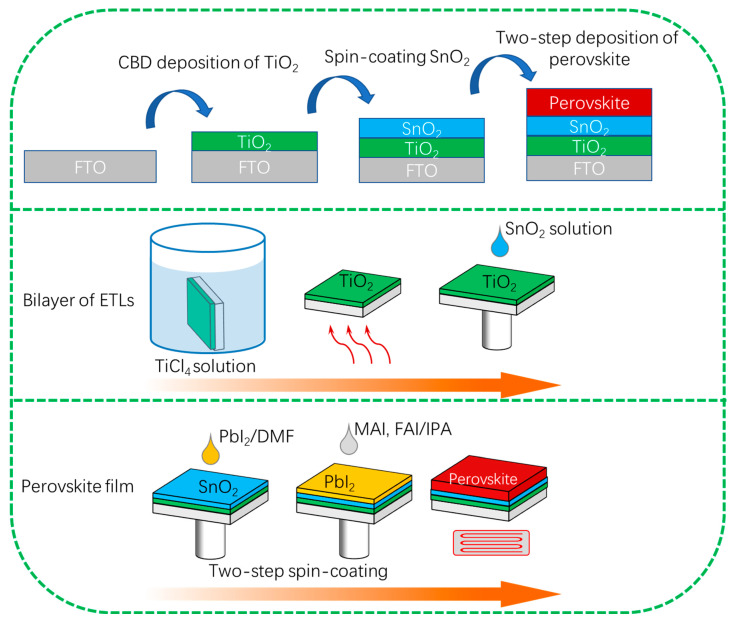
Schematic illustration of the bilayer of ETLs (TiO_2_ and SnO_2_ films) and perovskite films fabricated by chemical bath deposition and spin-coating.

Device Fabrication: The cleaned fluorine-doped tin oxide (FTO) substrates are treated using UV-ozone for 60 min. Then, the TiO_2_ thin layer was prepared by using the CBD method and the SnO_2_ thin layer was fabricated with spin-coating technologies, as shown in Figure 1. First, 2 M aqueous TiCl_4_ mother solution was prepared by dropping TiCl_4_ into distilled water. During the preparation, the mother solution was continuously stirred at a low temperature of around 0 °C. The as-prepared TiCl_4_ mother solution was stored in the refrigerator (<10 °C). Second, the as-prepared TiCl_4_ mother solution was diluted to a 0.2 M TiCl_4_ solution. The cleaned FTO substrates were placed vertically in the glassware. Then, 300 mL of 0.2 M TiCl_4_ solution was poured into the glassware. The glassware was put into an oven with a temperature of 75 °C. After 1 h heating, the glassware was taken out followed by rinsing the FTO substrates several times using distilled water. Finally, the FTO substrates were annealed at a high temperature of 450 °C for 30 min. The FTO substrates were washed by the acetone, distilled water, and ethyl alcohol for 20 min, respectively. Before the deposition of TiO_2_ thin films, the FTO substrates are treated by using UV-ozone for 60 min. SnO_2_ films were prepared by spin-coating Alfa Aesar SnO_2_ (diluted by H_2_O to 3%) at a speed of 3500 rpm for 30 s. The perovskite films were deposited by a two-step spin-coating method. Specifically, 1.35 M PbI_2_ and 0.0675 M CsI were dissolved in organic solvent (DMF/DMSO = 19:1). The PbI_2_ precursor solution was stirred at a temperature of 70 °C for 60 min. The mixed MAFA based organic cation precursor solution was prepared by dissolving 200 mg FAI, 100 mg MAI, 25 mg MABr and 25 mg MACl dissolved in 5 mL isopropanol. The PbI_2_ precursor solution was first spin-coated at a speed of 3000 rpm for 30 s. The MA/FA cation solution was spin-coated at 3000 rpm for 30s. After annealing at 150 °C for 10 min, the perovskite film of Cs_0.05_FA_0.54_MA_0.41_Pb(I_0.98_Br_0.02_)_3_ was obtained. The hole transport layer of the spiro-OMeTAD film was deposited by spin-coating the spiro-OMeTAD solution at a speed of 3500 rpm for 25 s. Finally, 80 nm Au film was deposited as a counter electrode by thermal evaporation.

Device Characterization: The diffraction data of perovskites are collected by using a Bruker D8 Discover diffractometer (Bruker AXS) from 10° to 60°. Surface and cross-section morphology images are recorded by a scanning electron microscope (SEM) (Helios NanoLab G3). The TRPL results were collected by using the Hamamatsu equipment which can provide an excitation wavelength of 450 nm. The photoluminescence (PL) spectra were acquired by a JASCO FP-8500 spectrometer with an excitation wavelength of 450 nm. The current-voltage (J-V) measurements were performed under one sun illumination (AM1.5G, 100 mW/cm^2^) by using a Keithley 2420. The devices were test by using a metal shadow mask with a dimension of 0.3 × 0.3 cm^2^. The EQE spectra of the devices were characterized by using Oriel IQE 200 equipment.

## 3. Results and Discussion

**Figure 2:**
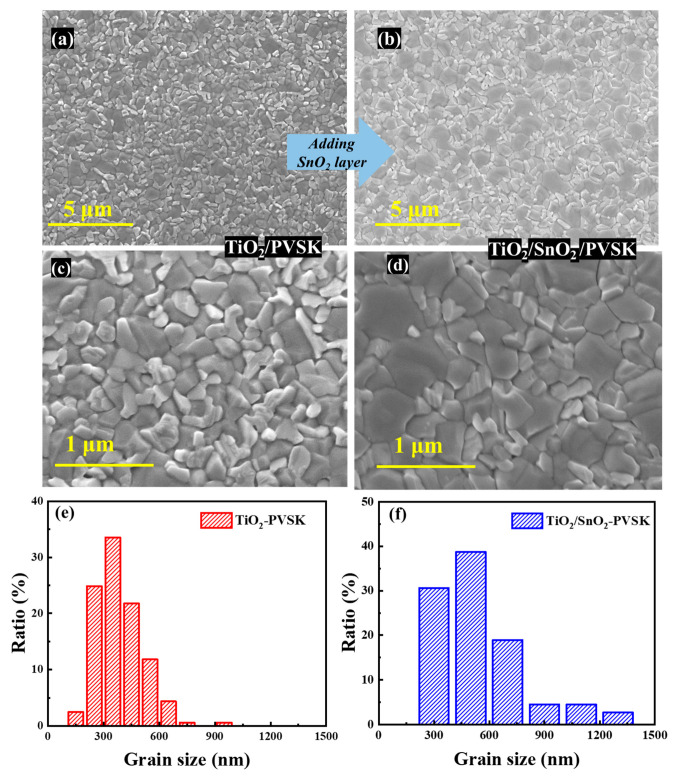
(**a**) Top-view SEM images of monolayer TiO_2_-ETL PSCs and bilayer TiO_2_/SnO_2_-ETL PSCs (**a**–**d**), and their corresponding statistic of grain size (**e**,**f**).

Figure 2a–d shows the top-view SEM images of the perovskite films fabricated on the TiO_2_ and TiO_2_/SnO_2_ substrates, which clearly shows a larger grain size of perovskite thin film based on the TiO_2_/SnO_2_ substrates, compared with that on the TiO_2_ substrates, with an average value changing from ~380 nm to ~540 nm, which can be verified by the statistics of perovskite grain size based on the TiO_2_ and TiO_2_/SnO_2_ substrates, as presented in Figure 2e,f. As is well-known, the commercial SnO_2_ colloid precursor is stabilized by incorporating potassium hydroxide (KOH) [24]. The presence of K ion in the SnO_2_ will diffuse into the perovskite thin film during the annealing process, which greatly enhances the crystallinity of perovskite grains, and reduces the hysteresis of resultant devices [25,26,27].

**Figure 3:**
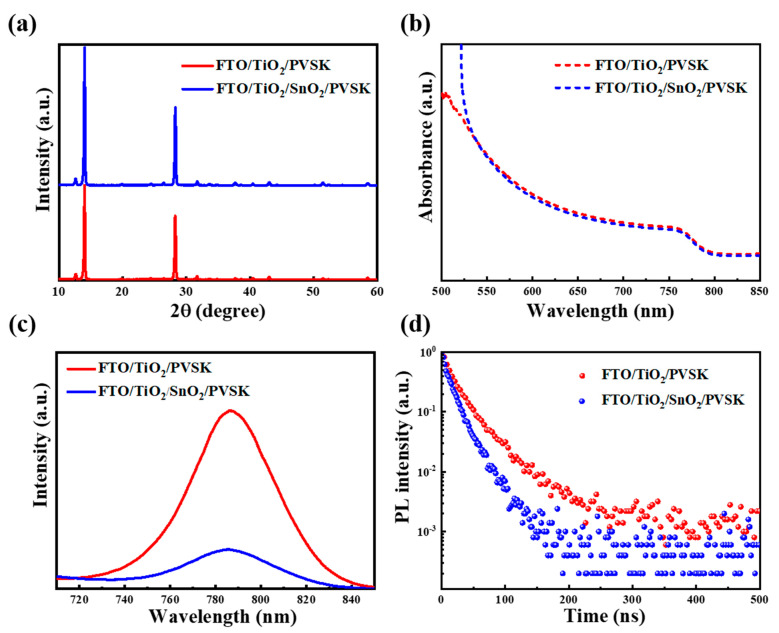
(**a**) XRD patterns, (**b**) absorption spectra, (**c**) PL spectra and (**d**) TRPL curves of the perovskite films deposited on FTO/TiO_2_ and FTO/TiO_2_/SnO_2_ substrates.

Furthermore, the phase structure of perovskite thin film deposited on the TiO_2_ and TiO_2_/SnO_2_ substrates was investigated by X-ray diffraction (XRD), as presented in Figure 3a. The increase of XRD intensity (on the TiO_2_/SnO_2_ substrate) verifies that the improved crystallinity of perovskites is accomplished with high absorption in a short-wavelength region (as shown in Figure 3b).

In addition, the steady-state photoluminescence (PL) and time-resolved photoluminescence (TRPL) experiments were carried out to investigate carrier transport behavior. As seen in Figure 3c,d, the faster PL quenching of the perovskite thin film on the TiO_2_/SnO_2_ substrate indicates an enhanced electron extraction capability [28]. Moreover, the lifetimes of the corresponding perovskite thin films were fitted by a biexponential decay function [29,30]. The lifetime of the TiO_2_/SnO_2_-based sample is 15.4 ns, which is shorter than that of the TiO_2_-based sample (22.2 ns), indicating a faster carrier extraction from the perovskite thin film to TiO_2_/SnO_2_ electron transport layer [31].

**Figure 4:**
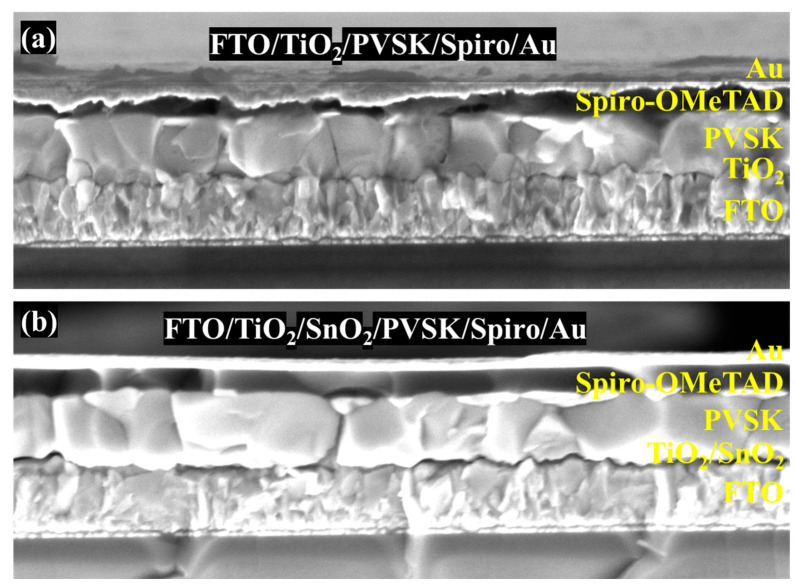
Cross-section images of PSCs in (**a**) TiO_2_-PSCs and (**b**) TiO_2_/SnO_2_ PSCs.

**Figure 5:**
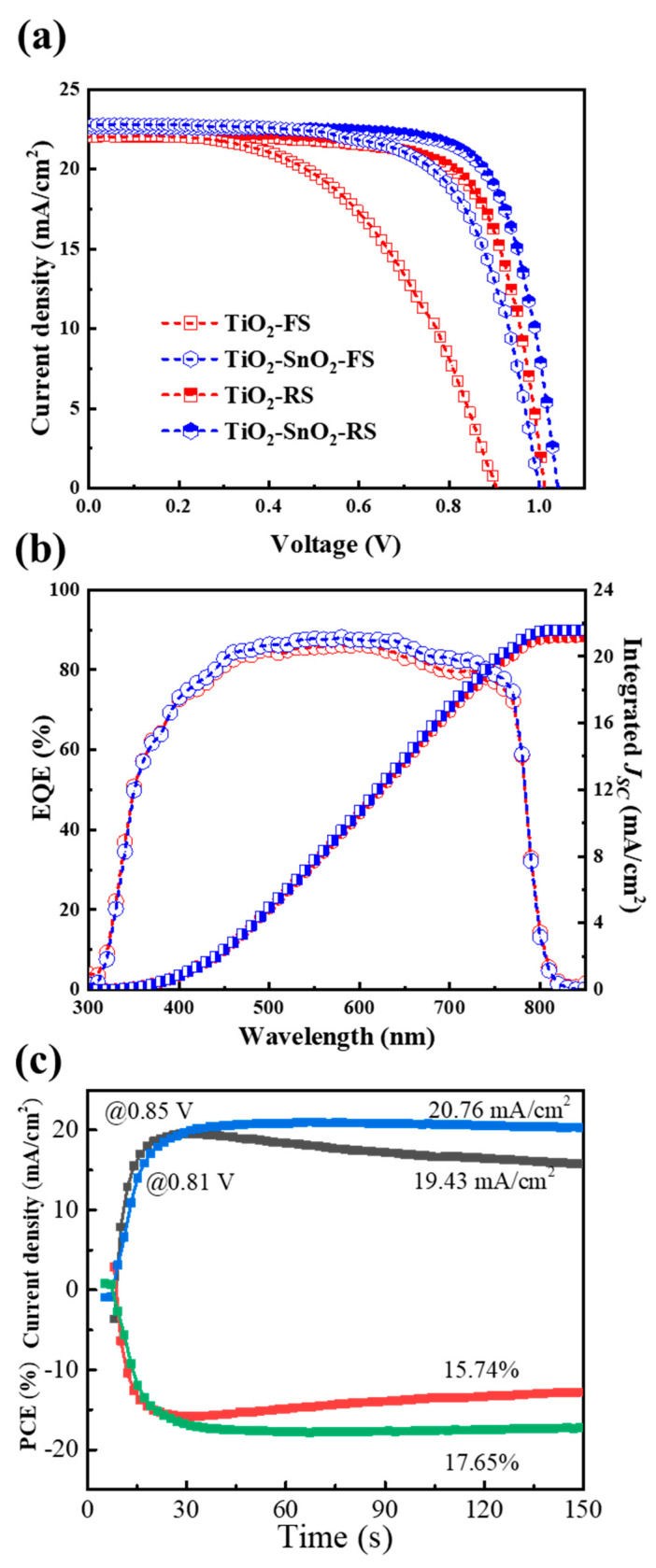
(**a**) Measured current density-voltage curves of champion devices. (**b**) Corresponding EQE spectra and their integrated current density. (**c**) Stable output of perovskite solar cells based on based on TiO_2_ and TiO_2_/SnO_2_ ETLs.

**Figure 6:**
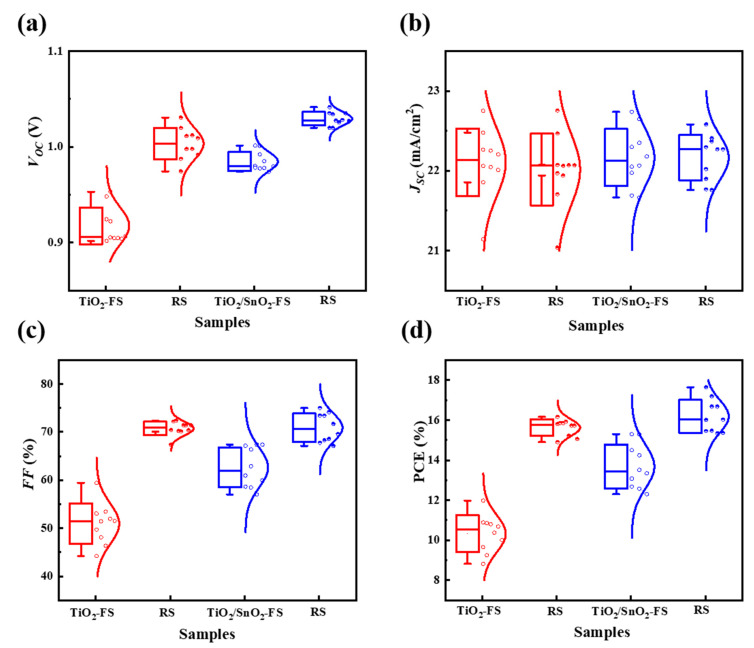
Statistical distribution of TiO_2_ and TiO_2_/SnO_2_ based PSCs (10 devices). (**a**) V_OC_, (**b**) J_SC_, (**c**) FF and (**d**) PCE.

Figure 4a,b shows the cross-section SEM images of devices fabricated on TiO_2_ and TiO_2_/SnO_2_ substrates. The uniform and dense perovskite absorber layers not only ensure the light harvest, but also effectively impede the carrier recombination in the devices. The current density-voltage (J-V) curves of the devices were measured under standard AM 1.5 G illumination and are shown in Figure 5a and Table 1, while the key performance parameters of open-circuit voltage (V_OC_), short-circuit current (J_SC_), fill factor (FF), power conversion efficiency (PCE) and their statistical analyses are displayed in Figure 6a–d and Table 2, respectively. The PCE of 16.16% (V_OC_ = 1.012 V, J_SC_ = 22.06 mA/cm^2^ and FF = 72.4%) and 10.37% (V_OC_ = 0.905 V, J_SC_ = 22.06 mA/cm^2^ and FF = 51.9%) under reverse scan (RS) and forward scan (FS) indicate large hysteresis in the TiO_2_-based devices. In contrast, the high PCE of 17.64% (V_OC_ = 1.041 V, J_SC_ = 22.58 mA/cm^2^ and FF = 75.0%) and 15.29% (V_OC_ = 1.001 V, J_SC_ = 22.73 mA/cm^2^ and FF = 67.2%) under RS and FS were obtained for TiO_2_/SnO_2_-based solar cells. The improved efficiency of TiO_2_/SnO_2_-based solar cells can be attributed to a higher crystallinity of perovskite grains, which enhances light capture and reduces the defects at grain boundaries [14,25]. The EQE spectra of the corresponding devices were presented in Figure 5b. The improved EQE in the short wavelength in terms of TiO_2_/SnO_2_-based device indicates faster carrier extraction and reduced recombination at the TiO_2_/SnO_2_/PVSK interface [32]. Similarly, the enhanced EQE at the long wavelength region also suggests that reduced defects and carrier recombination in the perovskite bulk film, which can be explained by the enlarged grain size and improved crystallinity of the perovskite grains [32]. As a result, the integrated J_SC_ from EQE of the TiO_2_/SnO_2_-based device is 21.59 mA/cm^2^, which is higher than that of the TiO_2_ based device (21.17 mA/cm^2^). Furthermore, the TiO_2_/SnO_2_-based device exhibited a stable output (under initial maximum power point (MPP) voltage) with a PCE of 17.65%. In contrast, the TiO_2_ based solar cell shows a poor output under MPP, yielding a low PCE of 15.74% (Figure 5c). More importantly, the hysteresis (hysteresis index (HI) = PCE_RS_/PCE_FS_) of the TiO_2_/SnO_2_-based devices is also reduced, compared to TiO_2_-based devices [32,33,34]. The HI of the TiO_2_-based PSC is 1.56, which is decreased to 1.15 by incorporating SnO_2_ into devices to construct the TiO_2_/SnO_2_ bilayer ETL. Compared to TiO_2_ based devices with a large hysteresis of 1.51, the improved efficiency and reduced HI of 1.18 for TiO_2_/SnO_2_-based PSCs indicates the bilayer ETL can improve the reproducible fabrication and the device performance.

## 4. Conclusions

In summary, we developed a bilayer electron transport layer by combining CBD-TiO_2_ and spin-coated SnO_2_ in the perovskite solar cells. The TiO_2_/SnO_2_ bilayer ETLs provide not only a compact electron transport layer, but also accelerate the carrier transport in the solar cells. Furthermore, the presence of K ion from SnO_2_ can greatly improve the crystallinity of perovskite thin film and significantly reduce the hysteresis of resultant devices. Compared with the TiO_2_-based solar cells, the TiO_2_/SnO_2_-based solar cells demonstrate a higher PCE of 17.64% and a lower hysteresis index. These results highlight the potential fabrication of the TiO_2_/SnO_2_ bilayer electron transport layers and will be a beneficial strategy to fabricate a high-quality perovskite thin film solar cell.

## Figures and Tables

**Table 1:** Photovoltaics parameters of PSCs based on TiO_2_ and TiO_2_/SnO_2_ ETLs.

Sample	Scan Direction	V_oc_ (V)	J_sc_ (mA/cm^2^)	FF	PCE (%)	HI
TiO_2_/SnO_2_ PSC	FS.	1.001	22.73	0.672	15.29	1.18
	RS.	1.041	22.58	0.750	17.64	
TiO_2_-PSCs	FS.	0.905	22.06	0.519	10.37	1.51
	RS.	1.012	22.06	0.724	16.16	

**Table 2:** Average photovoltaics parameters of PSCs based on TiO_2_ and TiO_2_/SnO_2_ ETLs.

Sample	Scan Direction	V_oc_ (V)	J_sc_ (mA/cm^2^)	FF	PCE (%)
TiO_2_/SnO_2_ PSC	FS.	0.985 ± 0.010	22.17 ± 0.34	0.626 ± 0.039	13.68 ± 1.04
	RS.	1.029 ± 0.007	22.16 ± 0.27	0.709 ± 0.028	16.18 ± 0.78
TiO_2_-PSCs	FS.	0.917 ± 0.018	22.11 ± 0.40	0.5090 ± 0.040	10.33 ± 0.88
	RS.	1.003 ± 0.016	22.01 ± 0.43	0.707 ± 0.014	15.61 ± 0.40

## Data Availability

The data is available on reasonable request from the corresponding author.
